# Supplementary material for: A Single-Cell and Spatial 3D Multi-omic Atlas of Developing Human Basal Ganglia and Inhibitory Neurons
Source: bioRxiv. 2026 Jan 29:2026.01.28.702385. Preprint. [Version 1] doi: 10.64898/2026.01.28.702385 (PMC12874046; doi:10.64898/2026.01.28.702385)
Supplement: 1 — Figure S1. Characterization of the developing basal ganglia and inhibitory neurons using snm3C-seq and spatial transcriptomics, related to Figure 1. Figure S2. Comparison of development dynamics across MSN subtypes and interneurons. Related to Figure 2. Figure S3. Chromatin changes across developmental stages. Related to Figure 3. Figure S4. Epigenomic changes in OPC/ODC during development. Related to Figure 4. Figure S5. Characterize the developing basal ganglia using highly multiplexed spatial transcriptomic and chromatin+RNA MERFISH. Figure S6. Epigenomic signatures of cortical arealization in developing human brains. Related to Figure 6. Figure S7. Regulatory landscapes of developing basal ganglia and inhibitory neurons. Related to Figure 7. [file NIHPP2026.01.28.702385V1-supplement-1.pdf]

bioRxiv preprint doi: https://doi.org/10.64898/2026.01.28.702385; this version posted January 29, 2026. The copyright holder for this preprint (which was not certified by peer review) is the author/funder, who has granted bioRxiv a license to display the preprint in perpetuity. It is made available under aCC-BY 4.0 International license.

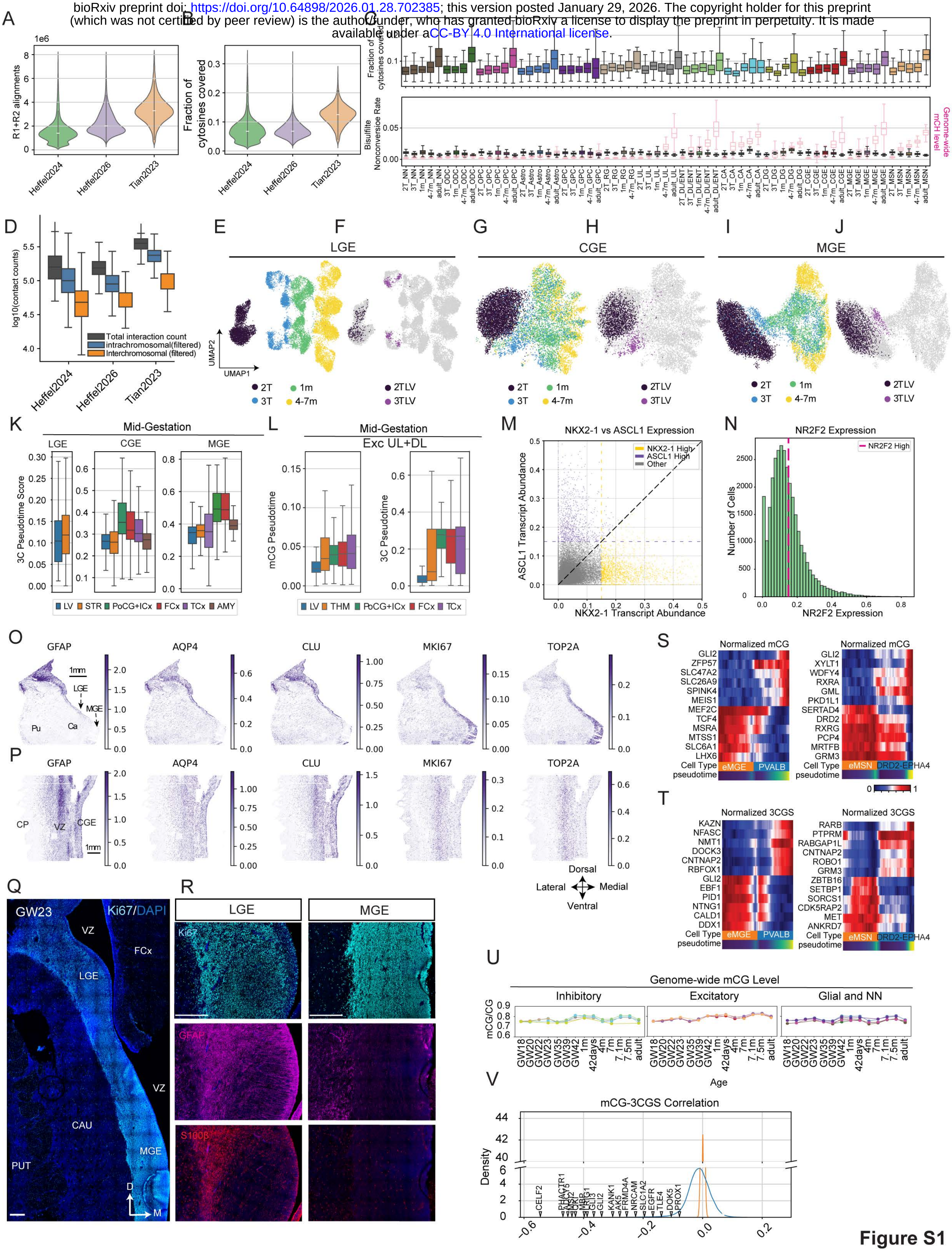

Figure S1

bioRxiv preprint doi: https://doi.org/10.64898/2026.01.28.702385; this version posted January 29, 2026. The copyright holder for this preprint (which was not certified by peer review) is the author/funder, who has granted bioRxiv a license to display the preprint in perpetuity. It is made available under aCC-BY 4.0 International license.

Figure S1. Characterization of the developing basal ganglia and inhibitory neurons using snm3C-seq and spatial transcriptomics, related to Figure 1. (A) The number of uniquely mapped methylome reads in each dataset integrated in this study. (B) The fraction of cytosines covered by cytosine positions in each dataset integrated in this study. (C) Coverage (top), bisulfite non-conversion rate (bottom), and mCH level (bottom) for major cell groups for each age group. (D) The number of total, intrachromosomal, and interchromosomal chromatin contacts in each dataset integrated in this study. (E-F) UMAP dimensionality reduction of LGE-derived cells labeled for age groups (E) or cells dissected from LV (F). (G-H) UMAP dimensionality reduction of CGE-derived cells labeled for age groups (G) or cells dissected from LV (H). (I-J) UMAP dimensionality reduction of MGE-derived cells labeled for age groups (I) or cells dissected from LV (J). (K) Distributions of single-cell 3C pseudotime scores for GE-derived neurons across brain regions. (L) Distributions of single-cell mCG (left) and 3C (right) pseudotime scores for excitatory neurons across cortical areas. (M) Cells with high NKX2-1 expression were selected by requiring > 0.15 normalized counts and greater than the abundance of ASCL1 transcripts. Cells with high ASCL1 expression were selected by requiring > 0.15 normalized counts and greater than the abundance of the NKX2-1 transcript. (N) Cells with high NR2F2 expression were selected by requiring > 0.15 normalized counts. (O-P) Transcript abundance of astrocyte and proliferation markers on the anterior section (O) and the posterior section (P). (S) Normalized mCG for marker genes that distinguish eMGE and MGE-PVALB (left), or eMSN and DRD2-Matrix (right). Normalized 3CGS for marker genes that distinguish eMGE and MGE-PVALB (left), or eMSN and DRD2-Matrix (right). (Q-R) An anterior section of a 23 GW section was stained for a proliferation marker Ki67 (Q-R), GFAP (R), and S100B during the differentiation and maturation of inhibitory (left), excitatory (mid), and glial cells (right). (V) The correlation between gene body mCG and 3C Gene Score (3CGS) highlights the strong inverse correlation at cell-type marker genes.

bioRxiv preprint doi: https://doi.org/10.64898/2026.01.28.702385; this version posted January 29, 2026. The copyright holder for this preprint (which was not certified by peer review) is the author/funder, who has granted bioRxiv a license to display the preprint in perpetuity. It is made available under aCC-BY 4.0 International license.

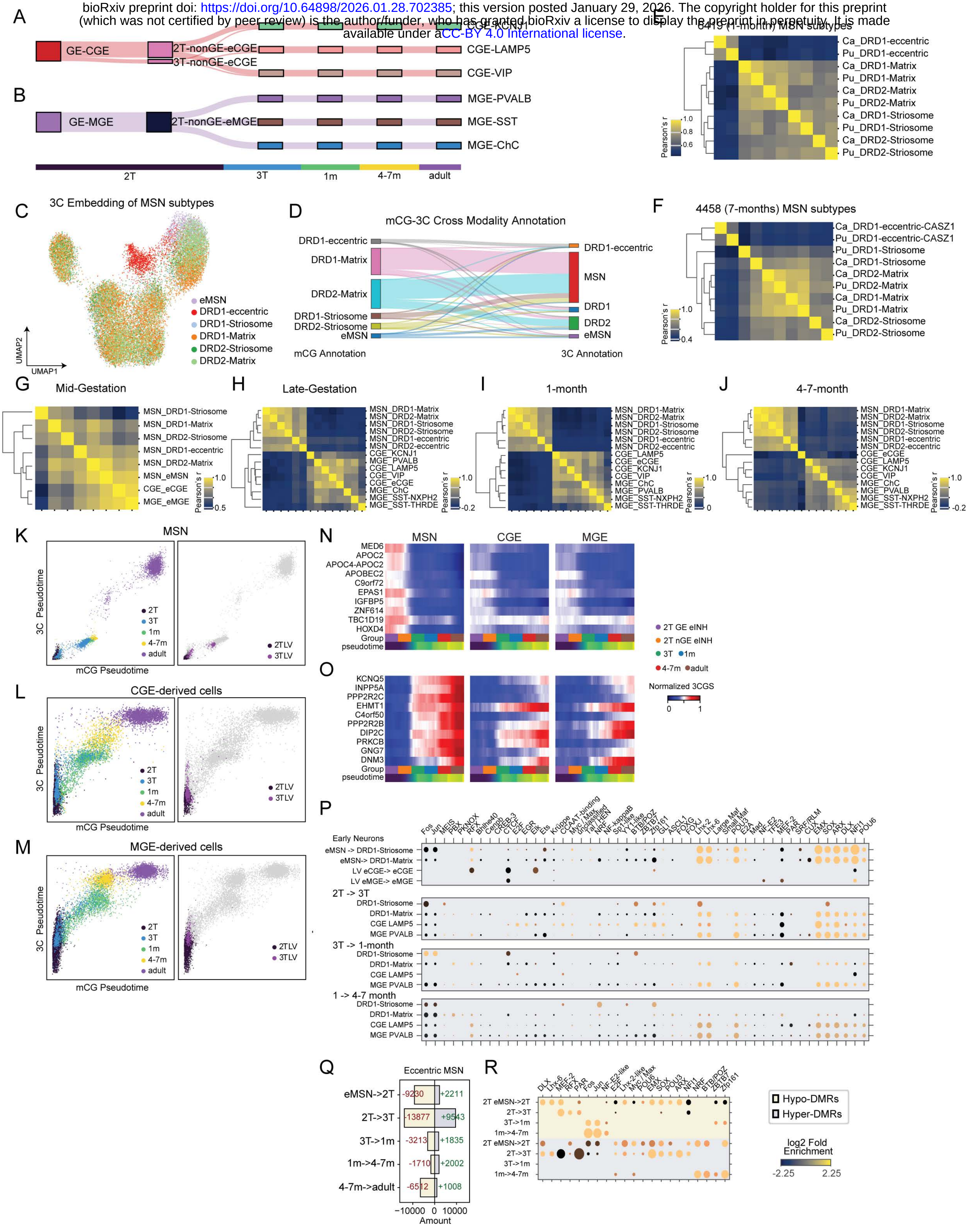

Figure S2

bioRxiv preprint doi: https://doi.org/10.64898/2026.01.28.702385; this version posted January 29, 2026. The copyright holder for this preprint (which was not certified by peer review) is the author/funder, who has granted bioRxiv a license to display the preprint in perpetuity. It is made available under aCC-BY 4.0 International license.

Figure S2. Comparison of development dynamics across MSN subtypes and interneurons. Related to Figure 2. (A-B) The specification of CGE (A) and MGE (B) subtypes in late-gestation. (C) UMAP embedding of MSN subtypes using 3C information. (D) mCG-3C cross-modality comparison of MSN neuron classifications. (E) Correlation matrix of MSN subtypes identified in an 1-month old donor (6419), computed using scaled gene body mCG. (F) Correlation matrix of MSN subtypes identified in a 7-month old donor (4458), computed using scaled gene body mCG. (G-J) Correlation matrices of inhibitory neuron subtypes identified in mid-gestational (G), late-gestational (H), 1-month-old (I), and 4–7-months-old (J) donors. (K-M) Comparison of pseudotime scores computed using mCG and 3C modalities across the differentiation of LGE-derived MSNs, and CGE- and MGE- derived interneurons, including cells derived from adult donors. (N-O) Scaled 3CGS of selected marker genes during the differentiation of LGE, CGE, and MGE-derived cell populations. The marker genes were selected for loss (N) and gain of 3CGS (O) during MSN differentiation. (P) TF binding motif enrichments in trajectory hyper-DMRs. (Q-R) Trajectory DMRs and associated TF enrichment (R) identified across the differentiation of eccentric MSNs.

bioRxiv preprint doi: https://doi.org/10.64898/2026.01.28.702385; this version posted January 29, 2026. The copyright holder for this preprint (which was not certified by peer review) is the author/funder, who has granted bioRxiv a license to display the preprint in perpetuity. It is made available under aCC-BY 4.0 International license.

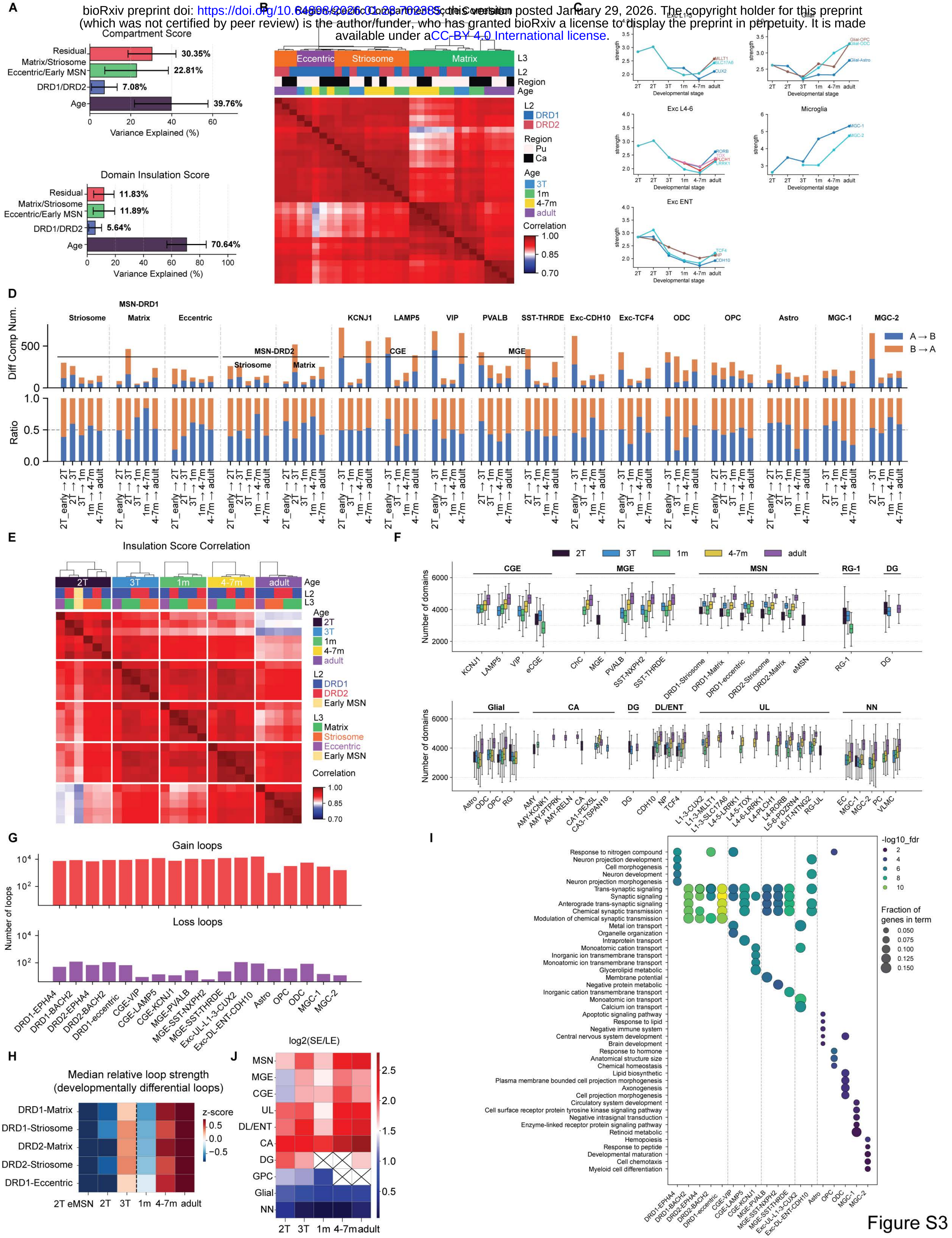

Figure S3

bioRxiv preprint doi: https://doi.org/10.64898/2026.01.28.702385; this version posted January 29, 2026. The copyright holder for this preprint (which was not certified by peer review) is the author/funder, who has granted bioRxiv a license to display the preprint in perpetuity. It is made available under aCC-BY 4.0 International license.

Figure S3. Chromatin changes across developmental stages. Related to Figure 3. (A) Variance partitioning based on ANOVA, showing the relative contributions of age, DRD identity, and MSN subtype to variation in 100-kb compartment scores and 25-kb insulation scores. (B) Genome-wide Pearson correlations of A/B compartment scores across MSN subtypes, stratified by Pu and Ca regions. (C) Developmental changes in compartment strength across excitatory neuronal lineages, glia, and microglia. (D) Number of differential compartments and the ratio of B-to-A and A-to-B compartment switches across cell types. (E) Genome-wide Pearson correlations of 25-kb bin insulation scores across MSN subtypes. (F) Number of domains identified across cell types and developmental stages. (G) Number of differential chromatin loops (gains and losses) across major developmental lineages. (H) Relative strength of developmentally regulated loops across MSN subtypes. (I) Gene Ontology (GO) enrichment analysis of genes associated with loop gains within ±2 kb of promoters. (J) Average $\log_2$ ratio of short- to long-range interactions across cell types and developmental stages.

bioRxiv preprint doi: https://doi.org/10.64898/2026.01.28.702385; this version posted January 29, 2026. The copyright holder for this preprint (which was not certified by peer review) is the author/funder, who has granted bioRxiv a license to display the preprint in perpetuity. It is made available under aCC-BY 4.0 International license.

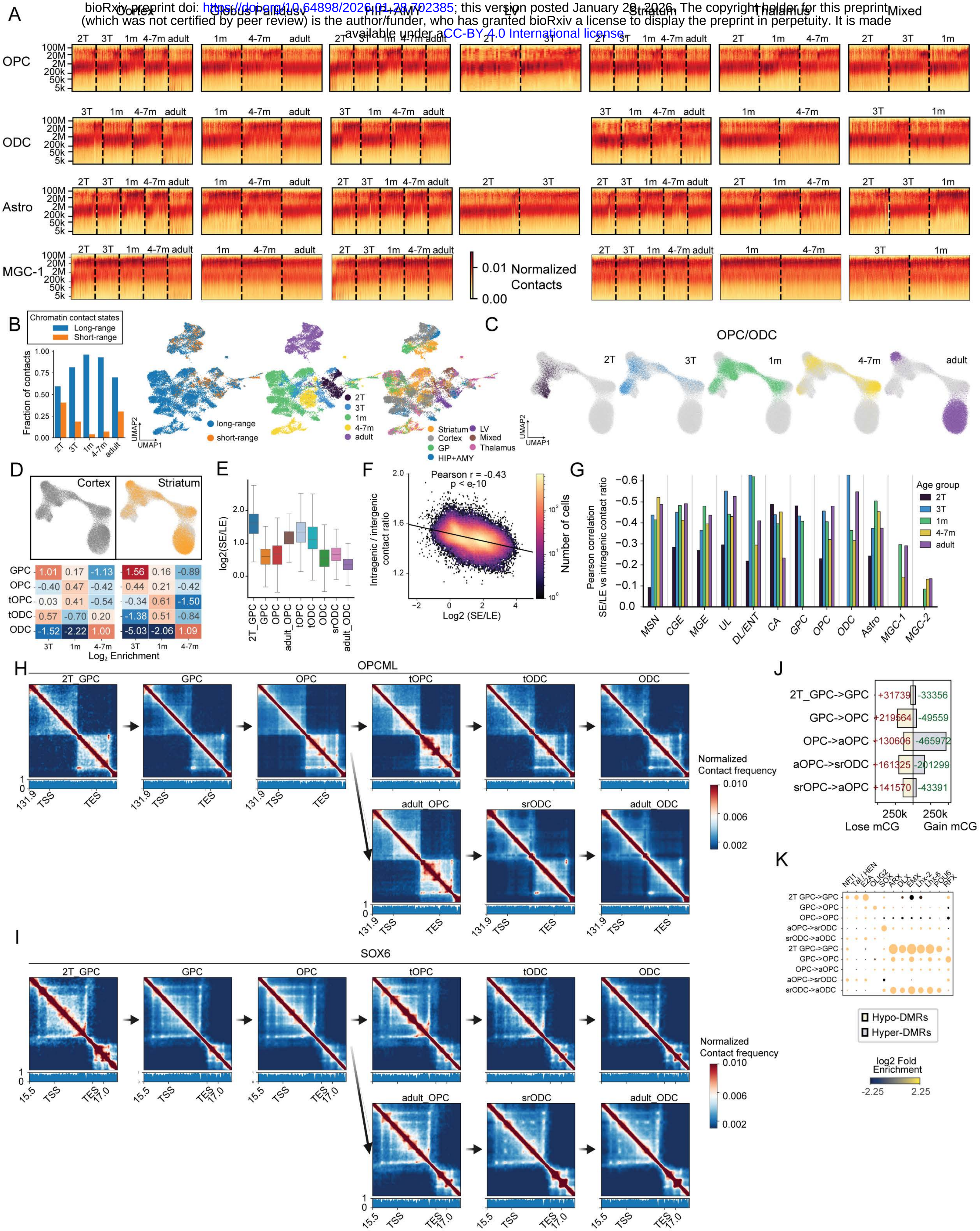

Figure S4

bioRxiv preprint doi: https://doi.org/10.64898/2026.01.28.702385; this version posted January 29, 2026. The copyright holder for this preprint (which was not certified by peer review) is the author/funder, who has granted bioRxiv a license to display the preprint in perpetuity. It is made available under aCC-BY 4.0 International license.

Figure S4. Epigenomic changes in OPC/ODC during development. Related to Figure 4. (A) Heatmaps depict contact frequency by genomic distance in individual cells across developmental stages and brain regions for OPCs, ODCs, astrocytes, and microglia-1. (B) Proportion of short- and long-range chromatin interactions (SE/LE) in astrocytes across developmental stages. (C) UMAP embedding of the 3C modality across developmental stages for OPC and ODC cells. (D) Log_2 enrichment of OPC/ODC subtypes across developmental stages in cortex and striatum. Enrichment was calculated as the ratio of the proportion of each cell type at a given developmental stage to its overall frequency across all stages. (E) log_2(SE/LE) across OPC and ODC subtypes. (F-G) Correlation between the log_2(SE/LE) and the intragenic/intergenic contact ratio across all cells (F) and within major cell types across developmental stages (G). (H-I) Local chromatin confirmation dynamics at OPCML and SOX6 during the differentiation of ODC in infant and adult brains. (J-K) Trajectory-DMRs (J) and TF binding motifs (K) identified across the differentiation of ODC in adult brains.

bioRxiv preprint doi: https://doi.org/10.64898/2026.01.28.702385; this version posted January 29, 2026. The copyright holder for this preprint (which was not certified by peer review) is the author/funder, who has granted bioRxiv a license to display the preprint in perpetuity. It is made available under aCC-BY 4.0 International license.

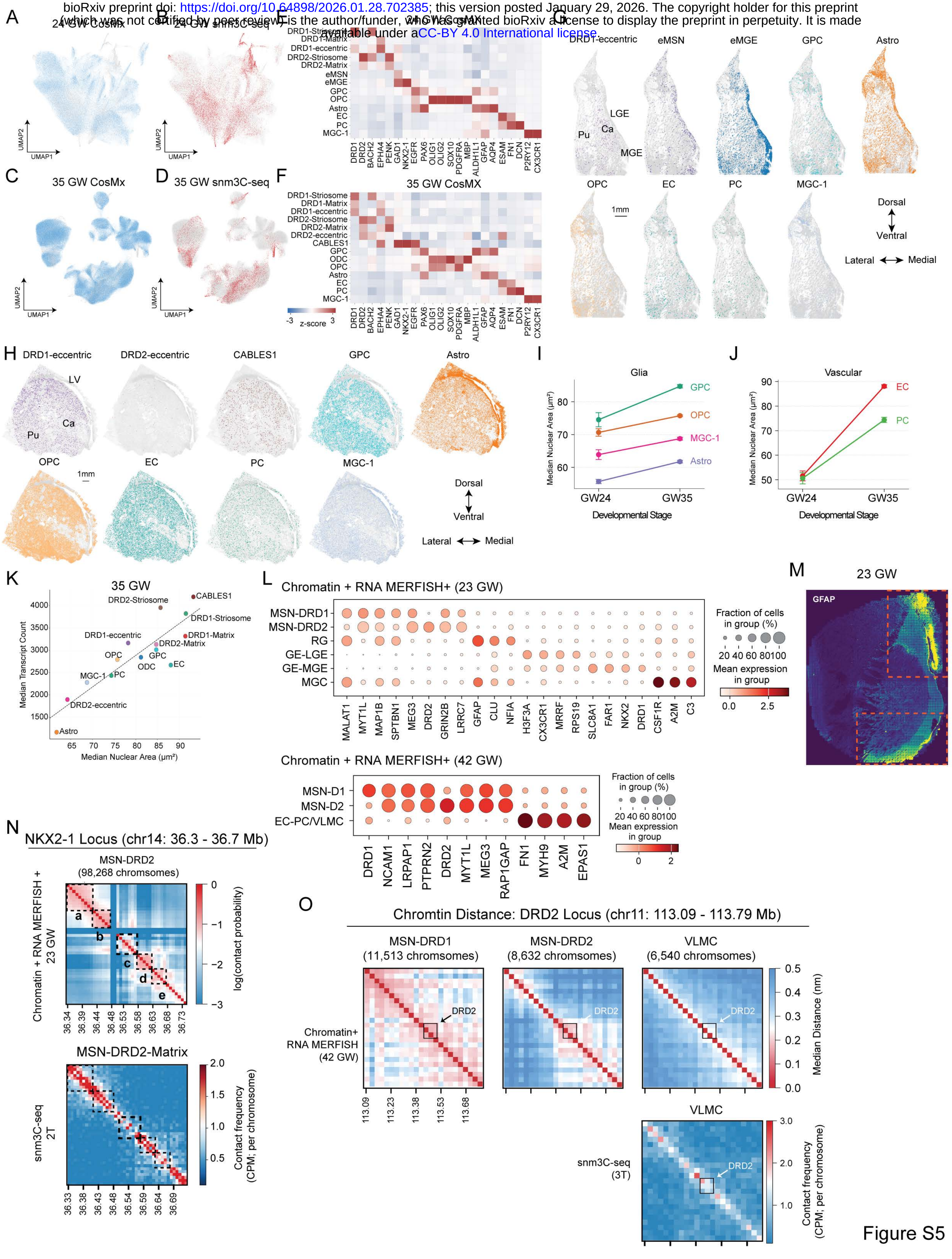

Figure S5

bioRxiv preprint doi: https://doi.org/10.64898/2026.01.28.702385; this version posted January 29, 2026. The copyright holder for this preprint (which was not certified by peer review) is the author/funder, who has granted bioRxiv a license to display the preprint in perpetuity. It is made available under aCC-BY 4.0 International license.

Figure S5. Characterize the developing basal ganglia using highly multiplexed spatial transcriptomic and chromatin+RNA MERFISH. Related to Figure 5. (A–D) UMAPs showing the integration of snm3C-seq cells with CosMx spatial transcriptomic profiles in the 24 GW and 35 GW samples. (E–F) Z-score expression values of marker genes used to identify major cell types in the integrated dataset. (G–H) Spatial maps of annotated cell populations in the 24 GW and 35 GW samples. (I–J) Nuclear area distributions for glial and other non-neuronal cell types across developmental stages. (K) Relationship between nuclear area and transcriptional activity in the 35 GW sample. (L) Marker-gene expression supporting major cell-type assignments in the combined chromatin tracing + MERFISH+ experiments. (M) Spatial map of GFAP expression in the 23 GW sample. (N) Chromatin tracing contact probability at the NKX2-1 locus across cell types in the 23 GW sample, alongside the corresponding snm3C-seq contact matrix. (O) Median chromatin distance from chromatin tracing at the DRD2 locus in the 42 GW sample, together with 3C contact maps for VLMC in the third trimester.

bioRxiv preprint doi: https://doi.org/10.64898/2026.01.28.702385; this version posted January 29, 2026. The copyright holder for this preprint (which was not certified by peer review) is the author/funder, who has granted bioRxiv a license to display the preprint in perpetuity. It is made available under aCC-BY 4.0 International license.

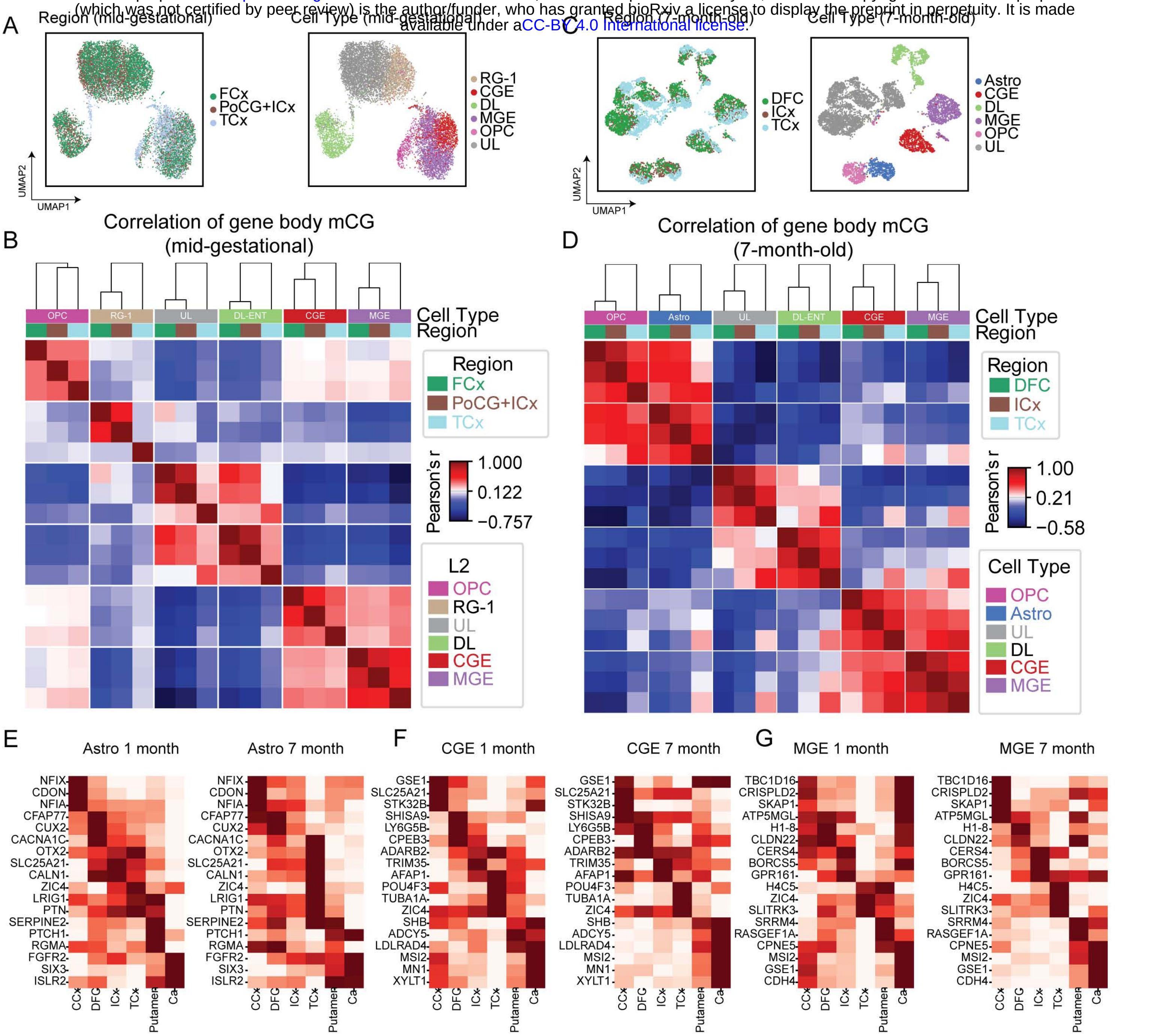

Figure S6

bioRxiv preprint doi: https://doi.org/10.64898/2026.01.28.702385; this version posted January 29, 2026. The copyright holder for this preprint (which was not certified by peer review) is the author/funder, who has granted bioRxiv a license to display the preprint in perpetuity. It is made available under aCC-BY 4.0 International license.

Figure S6. Epigenomic signatures of cortical arealization in developing human brains. Related to Figure 6. (A) mCG UMAP embedding of 23 GW old donor 2301 for major cell groups represented in the cortical regions colored by region (left) and lineage (right). (B) Pearson correlation of DFC, PoCG+ICx, and TCx across all major cell groups represented in these three dissections. (C) mCG UMAP embedding of 7 month old donor 4285 for major cell groups represented in the cortical regions, colored by region (left) and lineage (right). (D) Pearson correlation of DFC, ICx, and TCx across all L2 lineages represented in these three regions. (E-G) Top mCG genes shared by 1-month and 7-month samples for astrocyte (E), CGE-derived neurons (F), and MGE-derived neurons (G) regional specificity.

bioRxiv preprint doi: https://doi.org/10.64898/2026.01.28.702385; this version posted January 29, 2026. The copyright holder for this preprint (which was not certified by peer review) is the author/funder, who has granted bioRxiv a license to display the preprint in perpetuity. It is made available under aCC-BY 4.0 International license.

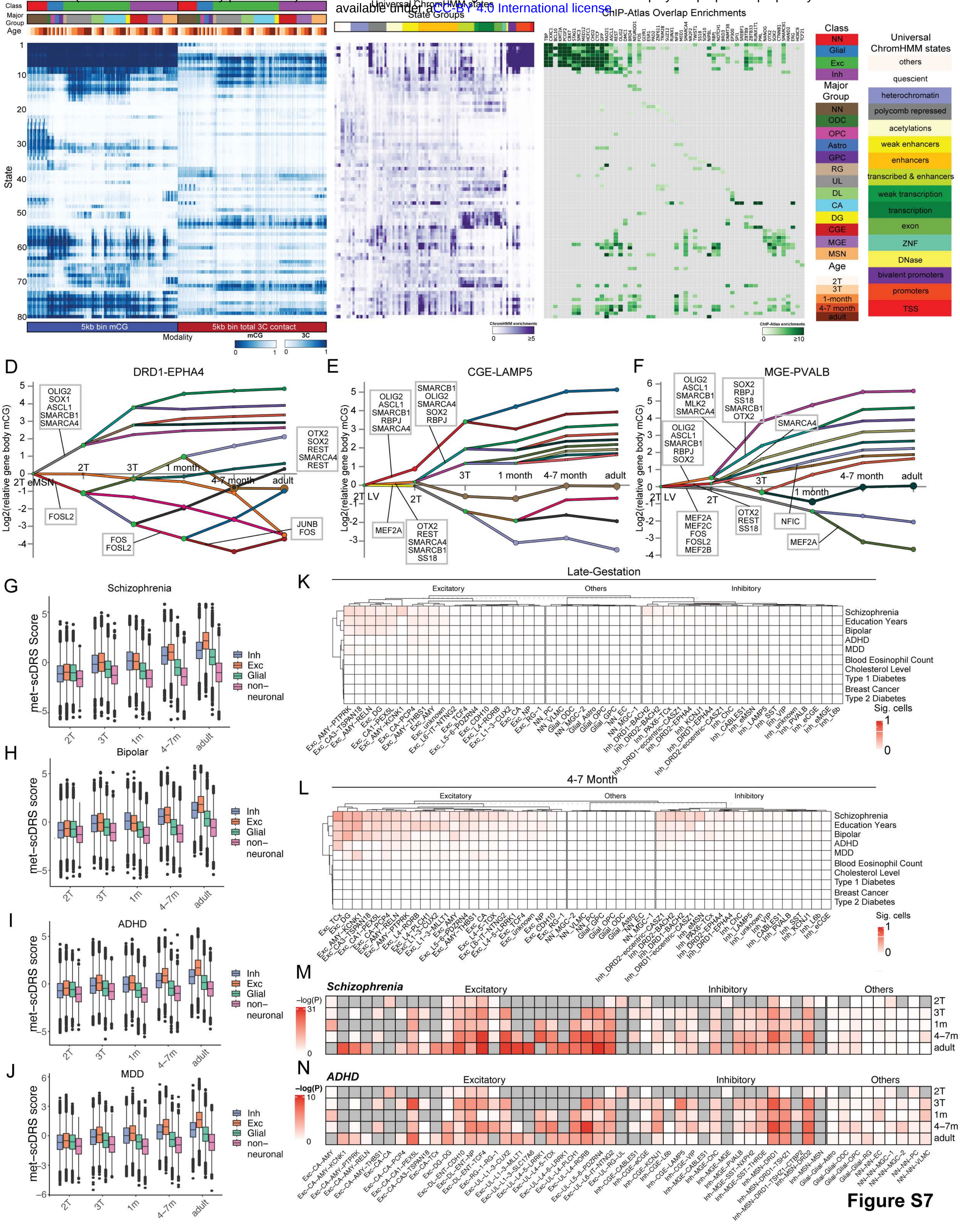

Figure S7

bioRxiv preprint doi: https://doi.org/10.64898/2026.01.28.702385; this version posted January 29, 2026. The copyright holder for this preprint (which was not certified by peer review) is the author/funder, who has granted bioRxiv a license to display the preprint in perpetuity. It is made available under aCC-BY 4.0 International license.

Figure S7. Regulatory landscapes of developing basal ganglia and inhibitory neurons. Related to Figure 7. (A) ChromHMM-based annotation of the human genome into 80 states (at a 5kbp resolution) based on the joint dynamic mCG and 3C patterns during human brain development. (B-C) Enrichment of universal ChromHMM states (B) and TF binding sites (C). For each state we selected the top-3 most enriched ChIP-Atlas experiments, then computed the median enrichment for each TF across the select experiments. (D-F) DREM reconstruction of transcriptional regulatory dynamics across the differentiation of matrix DRD1-expressing MSNs (D), CGE-LAMP5 interneurons (E), and MGE-PVALB interneurons (F). (G-J) Quantification of met-scDRS score in inhibitory neurons, excitatory neurons, glial cells, and non-neural cells, for SCZ (G), BIP (H), ADHD (I), and MDD (J). (K-L) Fraction of single cells showing significant met-scDRS score for selected neuropsychiatric and non-brain traits in the late-gestational (K) and 4-to-7 month old (L) human brains. (M-N) Enrichment of polygenic risks for SCZ (M) and ADHD (N) in pseudobulk methylome profiles determined by the LDSC partitioned heritability analysis.
